# Two new species of Hymenochaetaceae on *Dracaena cambodiana* from tropical China

**DOI:** 10.3897/mycokeys.80.63997

**Published:** 2021-05-05

**Authors:** Ping Du, Tian-Xu Cao, Ying-Da Wu, Meng Zhou, Zhan-Bo Liu

**Affiliations:** 1 College of Life Science and Technology, Yangtze Normal University, Chongqing 408100, China Yangtze Normal University Chongqing China; 2 China Fire and Rescue Institute, Beijing 102202, China China Fire and Rescue Institute Beijing China; 3 School of Ecology and Nature Conservation, Beijing Forestry University, Beijing 100083, China Beijing Forestry University Beijing China

**Keywords:** Phylogenetic analysis, taxonomy, wood-rotting fungi

## Abstract

Two new wood-rotting fungi in the family Hymenochaetaceae, *Fulvifomes dracaenicola***sp. nov.** and *Hymenochaete dracaenicola***sp. nov.**, are described and illustrated from tropical China based on morphological characteristics and molecular data. It is worth to mention that both of them grow on *Dracaena cambodiana* which is a kind of angiosperm tree distributed in tropical regions. *F. dracaenicola* is characterised by perennial, pileate, triquetrous basidioma with yellowish brown fresh pores which becoming honey yellow with silk sheening upon drying, a dimitic hyphal system in trama and monomitic in context, and subglobose basidiospores measuring 4.8–5 × 4–4.1 μm. *H. dracaenicola* is characterised by annual, resupinate basidioma with a clay buff hymenophore, a dimitic hyphal system, absence of tomentum and cortex, presence of subulate setae, absence of cystidia, presence of cystidioles and simple hyphidia, and oblong ellipsoid basidiospores measuring 5.2–5.8 × 2.5–2.8 µm. The phylogenetic analyses based on ITS + nLSU rDNA sequences confirm the placement of two new species respectively in *Fulvifomes* and *Hymenochaete*. Phylogenetically closely related species to the two new species are discussed.

## Introduction

*Fulvifomes* Murrill (Hymenochaetaceae, Hymenochaetales) was erected in 1914 and typified by *F. robiniae* (Murrill) Murrill (Murrill 1914). Wagner and Fischer (2002) thought that *Fulvifomes* comprises species with a dimitic hyphal system, absence of setae, and yellowish, thick-walled basidiospores. Hattori et al. (2014) provided a key to worldwide species of *Fulvifomes* and other species possibly belonging to *Fulvifomes*. Zhou (2014) treated *Aurificaria* D.A. Reid as a taxonomic synonym of *Fulvifomes* and transferred *Aurificaria indica* (Massee) D.A. Reid to *Fulvifomes*. However, *Fulvifomes indicus* (Massee) L.W. Zhou has a monomitic hyphal system, but he thought that the hyphal system might be not a stable character at the generic level within Hymenochaetaceae. Salvador-Montoya et al. (2018) redefined *Fulvifomes* and thought *Fulvifomes* should encompass species with a monomitic hyphal system in the context, a dimitic hyphal system in the trama. We agree with Zhou and Salvador-Montoya et al., and consider the genus *Fulvifomes* has a monomitic or dimitic hyphal system.

*Hymenochaete* Lév. (Hymenochaetaceae, Hymenochaetales) was erected in 1846 and typified by *H. rubiginosa* (Dicks.) Lév. (Léveillé 1846). Léger (1998) wrote a world monograph of *Hymenochaete* and provided a key of the genus. The genus comprises more than 120 species around the world (He and Dai 2012). *Hymenochaete* is characterised by annual to perennial, resupinate, effused-reflexed to pileate basidioma with smooth, tuberculate, lamellate, poroid or hydnoid hymenophores; a monomitic or dimitic hyphal system; presence of setae, and hyaline, thin-walled, narrowly cylindrical to globose basidiospores (Léger 1998; Parmasto 2001; He and Dai 2012).

During investigations on the diversity of wood-rotting fungi from China, five unknown specimens were collected from Hainan Province, and their morphology corresponds to the concepts of *Fulvifomes* and *Hymenochaete*. To confirm their affinity, phylogenetic analyses based on the ITS and nLSU rDNA sequences were carried out. Both morphological characteristics and molecular evidence demonstrated these five specimens represent two new species of Hymenochaetaceae, which we describe in the present paper.

## Materials and methods

### Morphological studies

Macro-morphological descriptions were based on field notes and dry herbarium specimens. Microscopic measurements and drawings were made from slide preparations of dried tissues stained with Cotton Blue and Melzer’s reagent following Dai (2010). Pores were measured by subjectively choosing as straight a line of pores as possible and measuring how many fit per mm. The following abbreviations are used: KOH = 5% potassium hydroxide, CB = Cotton Blue, CB– = acyanophilous, IKI = Melzer’s reagent, IKI– = neither amyloid nor dextrinoid, L = mean spore length (arithmetic average of all spores), W = mean spore width (arithmetic average of all spores), Q = variation in the L/W ratios between specimens studied, and n (a/b) = number of spores (a) measured from given number of specimens (b). In presenting spore size variation, 5% of measurements were excluded from each end of the range and this value is given in parentheses. Special color terms follow Anonymous (1969) and Petersen (1996). Herbarium abbreviations follow Thiers (2018). The studied specimens were deposited at the herbarium of the Institute of Microbiology, Beijing Forestry University (**BJFC**).

### Molecular studies and phylogenetic analysis

A CTAB rapid plant genome extraction kit (Aidlab Biotechnologies Co., Ltd., Beijing, China) was used to extract total genomic DNA from dried specimens following the manufacturer’s instructions with some modifications (Cui et al. 2019; Shen et al. 2019). ITS regions were amplified with primers ITS4 and ITS5 (White et al. 1990), and the nLSU with primers LR0R and LR7. The polymerase chain reaction (PCR) procedure for ITS was as follows: initial denaturation at 95 °C for 3 min, followed by 35 cycles at 94 °C for 40 s, 58 °C for 45 s, and 72 °C for 1 min, and a final extension of 72 °C for 10 min. The PCR procedure for nLSU was as follows: initial denaturation at 94 °C for 1 min, followed by 35 cycles at 94 °C for 30 s, 48 °C for 1 min, and 72 °C for 1.5 min, and a final extension of 72 °C for 10 min (Chen et al. 2015). The PCR products were purified and sequenced in the Beijing Genomics Institute, China, with the same primers used in the PCR reactions.

Phylogenetic trees were constructed using ITS and nLSU rDNA sequences, and phylogenetic analyses were computed with maximum likelihood (ML), maximum parsimony (MP), and Bayesian inference (BI) methods. Sequences of *Fulvifomes* were adopted mainly from ITS + nLSU tree topologies established by Liu et al. (2020). Sequences of *Hymenochaete* were adopted mainly from ITS + nLSU tree topologies established by He et al. (2017) and Rossi et al. (2020). New sequences generated in this study, along with reference sequences retrieved from GenBank (Table 1 and Table 2), were aligned by MAFFT 6 (Katoh and Toh 2008; http://mafft.cbrc.jp/alignment/server/) using the “G-INS-i” strategy and manually adjusted in BioEdit (Hall 1999). The data matrix was edited in Mesquite v3.04 software (Maddison and Maddison 2010). The sequence alignment was deposited at TreeBase (*Fulvifomes*, http://purl.org/phylo/treebase/phylows/study/TB2:S27995; submission ID 27995) and (*Hymenochaete*, http://purl.org/phylo/treebase/phylows/study/TB2:S27696; submission ID 27696). Sequences of *Phellinus laevigatus* (P. Karst.) Bourdot & Galzin and *P. populicola* Niemelä obtained from GenBank were used as outgroups of *Fulvifomes* to root trees following Ji et al. (2017) in the ITS + nLSU analysis. Sequences of *Hydnoporia tabacina* (Sowerby) Spirin, Miettinen & K.H. Larss. obtained from GenBank were used as outgroups of *Hymenochaete* to root trees following He et al. (2017) in the ITS + nLSU analysis.

**Table 1.** A list of species, specimens and GenBank accession numbers of sequences used in the phylogenetic analysis of *Fulvifomes*.

Taxa	Voucher	ITS	LSU
*Fomitiporella caryophylli*	CBS 448.76	AY558611	AY059021
*Fulvifomes centroamericanus*	JV 0611/III	KX960763	KX960764
*F. centroamericanus*	JV 0611/8P	KX960757	—
***F. dracaenicola***	**Dai 22093**	**MW559799**	**MW559804**
***F. dracaenicola***	**Dai 22097**	**MW559800**	**MW559805**
*F. fastuosus*	LWZ 20140731-13	KR905674	KR905668
*F. fastuosus*	LWZ 20140718-29	KR905673	—
*F. fastuosus*	Dai 18292	MH390411	MH390381
*F. grenadensis*	JV 1212/2J	KX960756	—
*F. grenadensis*	JV 1607/66	KX960758	—
*F. hainanensis*	Dai 11573	KC879263	JX866779
*F. halophilus*	XG 4	JX104705	JX104752
*F. halophilus*	JV 1502/4	MH390427	MH390392
*F. imbricatus*	LWZ 20140728-16	KR905677	KR905670
*F. imbricatus*	LWZ 20140729-25	KR905678	—
*F. imbricatus*	LWZ 20140729-26	KR905679	KR905671
*F. indicus*	Yuan 5932	KC879261	JX866777
*F. indicus*	O 25034	KC879262	KC879259
*F. krugiodendri*	JV 0904/1	KX960762	KX960765
*F. krugiodendri*	JV 0312/24.10J	KX960760	KX960766
*F. krugiodendri*	JV 1008/21	KX960761	KX960767
*F. merrillii*	—	JX484013	—
*F. nilgheriensis*	URM 3028	MH390431	MH390384
*F. nilgheriensis*	PPT152	MH048095	MH048085
*F. rimosus*	M 2392655	MH628255	MH628017
*F. robiniae*	CBS 211.36	AY558646	AF411825
*F. robiniae*	—	EF088656	—
*F. siamensis*	XG 2	JX104709	JX104756
*F. siamensis*	Dai 18309	MH390434	MH390389
*F.* sp.	PM 950703-1	EU035311	—
*F. squamosus*	CS385	MF479268	MF479265
*F. squamosus*	CS444	MF479269	MF479264
*F. submerrillii*	Dai 17911	MH390405	MH390371
*F. submerrillii*	Dai 17917	MH390406	MH390372
*F. thailandicus*	LWZ 20140731-1	KR905672	KR905665
*F. xylocarpicola*	MU 8	JX104676	JX104723
*Inocutis rheades*	—	AF237731	—
*Inonotus hispidus*	CBS 388.61	AY558602	—
*I. lloydii*	Dai 10809	MH390428	MH390378
*I. lloydii*	Dai 9642	MH390429	MH390379
*I. lloydii*	Dai 11978	MH390430	MH390380
*I. porrectus*	CBS 296.56	AY558603	AY059051
*I. rigidus*	Dai 17496	MH390432	MH390398
*I. rigidus*	Dai 17507	MH390433	MH390399
*Phellinotus neoaridus*	URM 80362	KM211294	KM211286
*P. piptadeniae*	URM 80766	KM211293	KM211285
*Phellinus laevigatus*	CBS 122.40	MH856059	MH867554
*P. populicola*	CBS 638.75	MH860960	MH872729
*Phylloporia crataegi*	Dai 18133	MH151191	MH165865
*P. ribis*	CBS 579.50	MH856765	MN240818

New species is shown in bold.

**Table 2.** A list of species, specimens and GenBank accession numbers of sequences used in the phylogenetic analysis of *Hymenochaete*.

Taxa	Voucher	ITS	LSU
*Hydnoporia tabacina*	He 390	JQ279610	JQ279625
*Hymenochaete acerosa*	He 338	JQ279543	JQ279657
*H. adusta*	He 207	JQ279523	KU975497
*H. angustispora*	Dai 17045	MF370592	MF370598
*H. angustispora*	Dai 17049	MF370593	MF370599
*H. anomala*	He 592	JQ279566	JQ279650
*H. asetosa*	Dai 10756	JQ279559	JQ279642
*H. attenuata*	He 28	JQ279526	JQ279633
*H. bambusicola*	He 4116	KY425674	KY425681
*H. berteroi*	He 1488	KU975459	KU975498
*H. biformisetosa*	He 1445	KF908247	KU975499
*H. boddingii*	MEH 66068	MN030343	MN030345
*H. boddingii*	MEH 69996	MN030341	MN030347
*H. boddingii*	MEH 66150	MN030344	MN030344
*H. borbonica*	CBS 731.86	MH862026	MH873716
*H. cana*	He 1305	KF438169	KF438172
*H. cinnamomea*	He 755	JQ279548	JQ279658
*H. colliculosa*	Dai 16427	MF370595	MF370602
*H. colliculosa*	Dai 16428	MF370596	MF370603
*H. colliculosa*	Dai 16429	MF370597	MF370604
*H. conchata*	MEH 70144	MF373838	—
*H. contiformis*	He 1166	KU975461	KU975501
*H. cruenta*	He 766	JQ279595	JQ279681
*H. cyclolamellata*	Cui 7393	JQ279513	JQ279629
*H. damicornis*	URM 84261	KC348466	—
*H. damicornis*	URM 84263	KC348467	—
*H. denticulata*	He 1271	KF438171	KF438174
***H. dracaenicola***	**Dai 22090**	**MW559797**	**MW559802**
***H. dracaenicola***	**Dai 22096**	**MW559798**	**MW559803**
*H. duportii*	AFTOL ID666	DQ404386	AY635770
*H. epichlora*	He 525	JQ279549	JQ279659
*H. floridea*	He 536	JQ279597	JQ279683
*H. fuliginosa*	He 1188	KU975465	KU975506
*H. fulva*	He 640	JQ279565	JQ279648
*H. globispora*	He 911	—	KU975508
*H. huangshanensis*	He 432	JQ279533	JQ279671
*H. hydnoides*	He 245	JQ279590	JQ279680
*H. innexa*	He 555	JQ279584	JQ279674
*H. legeri*	He 960	KU975469	KU975511
*H. longispora*	He 217	JQ279537	KU975514
*H. luteobadia*	He 8	JQ279569	KU975515
*H. macrochloae*	ARAN-Fungi 7079	MF990738	MF990743
*H. megaspora*	He 302	JQ279553	JQ279660
*H. minor*	He 933	JQ279555	JQ279654
*H. minuscula*	He 253	JQ279546	KU975516
*H. murina*	He 569	JQ716406	JQ716412
*H. muroiana*	He 405	JQ279542	KU975517
*H. nanospora*	He 475	JQ279531	JQ279672
*H. ochromarginata*	He 47	JQ279579	JQ279666
*H. odontoides*	Dai 11635	JQ279563	JQ279647
*H. orientalis*	He 4601	KY425677	KY425685
*H. parmastoi*	He 867	JQ780063	KU975518
*H. paucisetigera*	Cui 7845	JQ279560	JQ279644
*H. quercicola*	He 373	KU975474	KU975521
*H. rhabarbarina*	He 280	JQ279574	KY425688
*H. rheicolor*	Cui 8317	JQ279529	—
*H. rhododendricola*	He 389	JQ279577	JQ279653
*H. rubiginosa*	He 1049	JQ716407	JQ279667
*H. rufomarginata*	He 1489	KU975477	KU975524
*H. separabilis*	He 460	JQ279572	JQ279655
*H. setipora*	Cui 6301	JQ279515	JQ279639
*H. sharmae*	CAL 1535	KY929017	KY929018
*H. sharmae*	66088	MK588753	MK588836
*H. spathulata*	He 685	JQ279591	KU975529
*H. sphaericola*	He 303	JQ279599	JQ279684
*H. sphaerospora*	He 715	JQ279594	KU975531
*H. subferruginea*	Cui 8122	JQ279521	—
*H. subferruginea*	He 1598	KU975481	—
*H. tasmanica*	He 449	JQ279582	JQ279663
*H. tongbiguanensis*	He 1552	KF908248	KU975532
*H. tenuis*	He 779	JQ279538	JQ279641
*H. tropica*	He 574	JQ279587	JQ279675
*H. ulmicola*	He 864	JQ780065	KU975534
*H. unicolor*	He 468a	JQ279551	JQ279662
*H. verruculosa*	Dai 17047	—	MF370600
*H. verruculosa*	Dai 17052	MF370594	MF370601
*H. villosa*	He 537	JQ279528	JQ279634
*H. xerantica*	Cui 9209	JQ279519	JQ279635
*H. yunnanensis*	He 1447	KU975486	KU975538

New species is shown in bold.

Maximum parsimony analysis was applied to the ITS + nLSU dataset sequences. Approaches to phylogenetic analysis followed Song et al. (2016), and the tree construction procedure was computed in PAUP* version 4.0b10 (Swofford 2002). All characters were equally weighted and gaps were treated as missing data. Trees were inferred using the heuristic search option with tree bisection and reconnection (TBR) branch swapping and 1000 random sequence additions maxtrees were set to 5000, branches of zero length were collapsed, and all parsimonious trees were saved. Clade robustness was assessed using a bootstrap (BT) analysis with 1000 replicates (Felsenstein 1985). Descriptive tree statistics tree length (TL), Consistency Index (CI), Retention Index (RI), Rescaled Consistency index (RC), and Homoplasy Index (HI) were calculated for each maximum parsimonious tree (MPT) generated. Sequences were also analyzed using maximum likelihood (ML) with RAxML-HPC through the CIPRES Science Gateway (Miller et al. 2009; http://www.phylo.org). Branch support for ML analysis was determined by 1000 bootstrap replicates.

MrModeltest 2.3 (Posada and Crandall 1998; Nylander 2004) was used to determine the best-fit evolution model for the combined dataset for Bayesian Inference (BI). BI was performed using MrBayes v. 3.2.7a (Ronquist and Huelsenbeck 2003) with four simultaneous independent chains for two datasets, performing 3 million generations (*Fulvifomes*) and 5 million generations (*Hymenochaete*) until the split deviation frequency value < 0.01, and sampled every 1000^th^ generation. The first 25% sampled trees were discarded as burn-in, while the remaining ones were used to calculate Bayesian posterior probabilities (BPP) of the clades.

Branches that received bootstrap support for maximum likelihood (BS), maximum parsimony (BP), and Bayesian posterior probabilities (BPP) greater than 70% (BS), 50% (BP) and 0.95 (BPP) were considered as significantly supported, respectively. FigTree v1.4.2 (Rambaut 2012) was used to visualize the resulting tree.

## Results

### Phylogeny results

#### Fulvifomes

The combined ITS + nLSU dataset included sequences from 50 specimens representing 31 species (Table 1). The dataset had an aligned length of 1693 characters, of which 1013 (60%) were constant, 186 (11%) were variable but parsimony-uninformative, and 494 (29%) were parsimony-informative. MP analysis yielded two equally parsimonious trees (TL = 1841, CI = 0.546, RI = 0.712, RC = 0.389, HI = 0.454). The best model-fi for the ITS + nLSU dataset estimated and applied in the Bayesian analysis was GTR+I+G. Bayesian analysis and ML analysis resulted in a similar topology to the MP analysis, with an average standard deviation of split frequencies of 0.004578 (BI).

**Figure 1.**
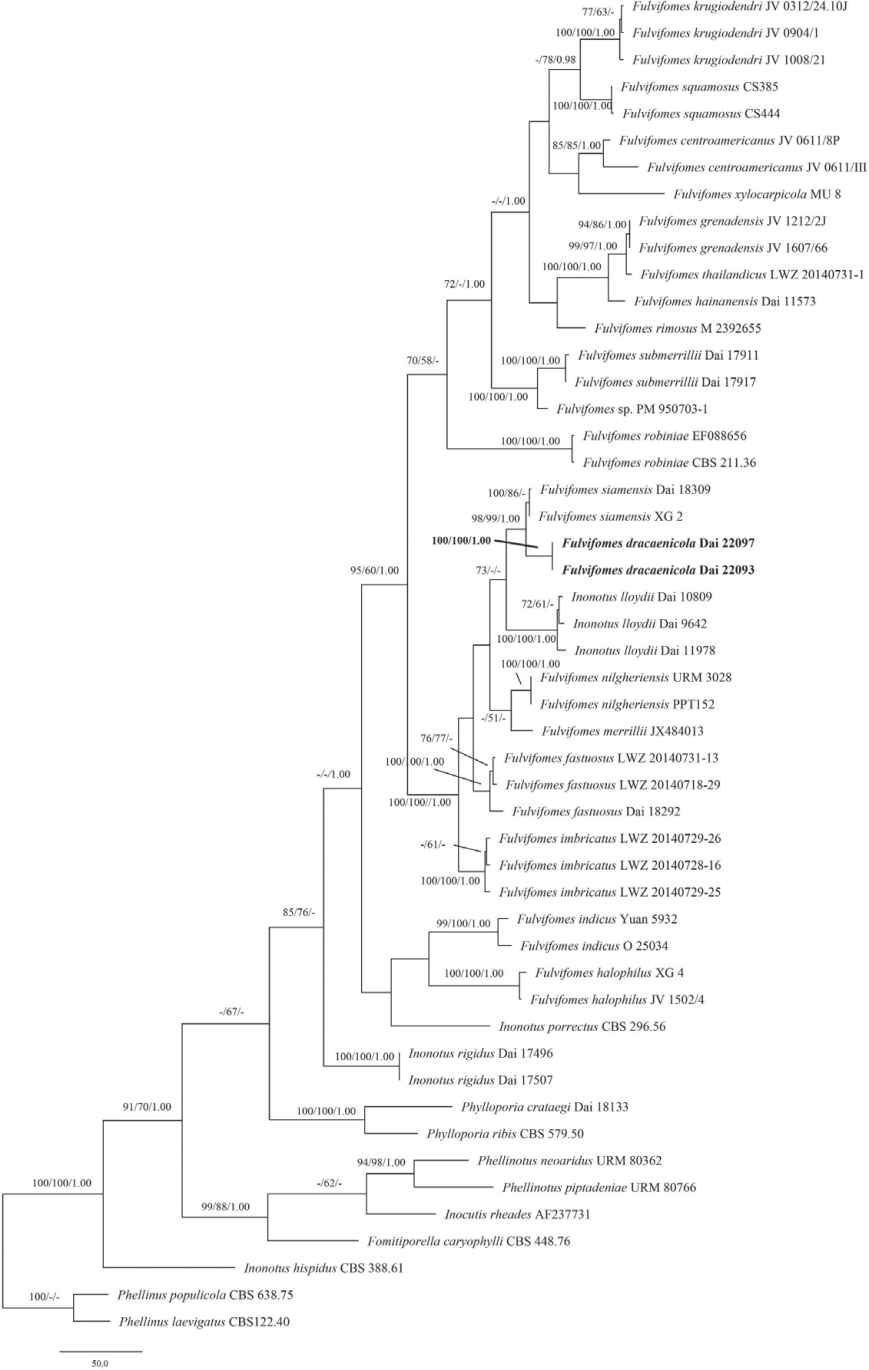
Phylogeny of *Fulvifomes* and related species by MP analysis based on combined ITS and nLSU rDNA sequences. Branches are labelled with maximum likelihood bootstrap > 70%, parsimony bootstrap proportions > 50%, and Bayesian posterior probabilities > 0.95, respectively. New species is in bold.

The phylogeny (Fig. 1) inferred from the ITS and nLSU sequences demonstrated that the new species *Fulvifomes dracaenicola* nested in the *Fulvifomes* clade. Moreover, two specimens of *F. dracaenicola* form a lineage with strong support (100% BP, 100% BS, 1.00 BPP, Fig. 1).

#### Hymenochaete

The combined ITS + nLSU dataset included sequences from 79 specimens representing 69 species (Table 2). The dataset had an aligned length of 2249 characters, of which 1486 (66%) were constant, 248 (11%) were variable but parsimony-uninformative, and 515 (23%) were parsimony-informative. MP analysis yielded 48 equally parsimonious trees (TL = 3261, CI = 0.365, RI = 0.619, RC = 0.226, HI = 0.635). The best model for the ITS + nLSU dataset estimated and applied in the Bayesian analysis was GTR+I+G. Bayesian analysis and MP analysis resulted in a similar topology to the ML analysis, with an average standard deviation of split frequencies of 0.009996 (BI).

**Figure 2.**
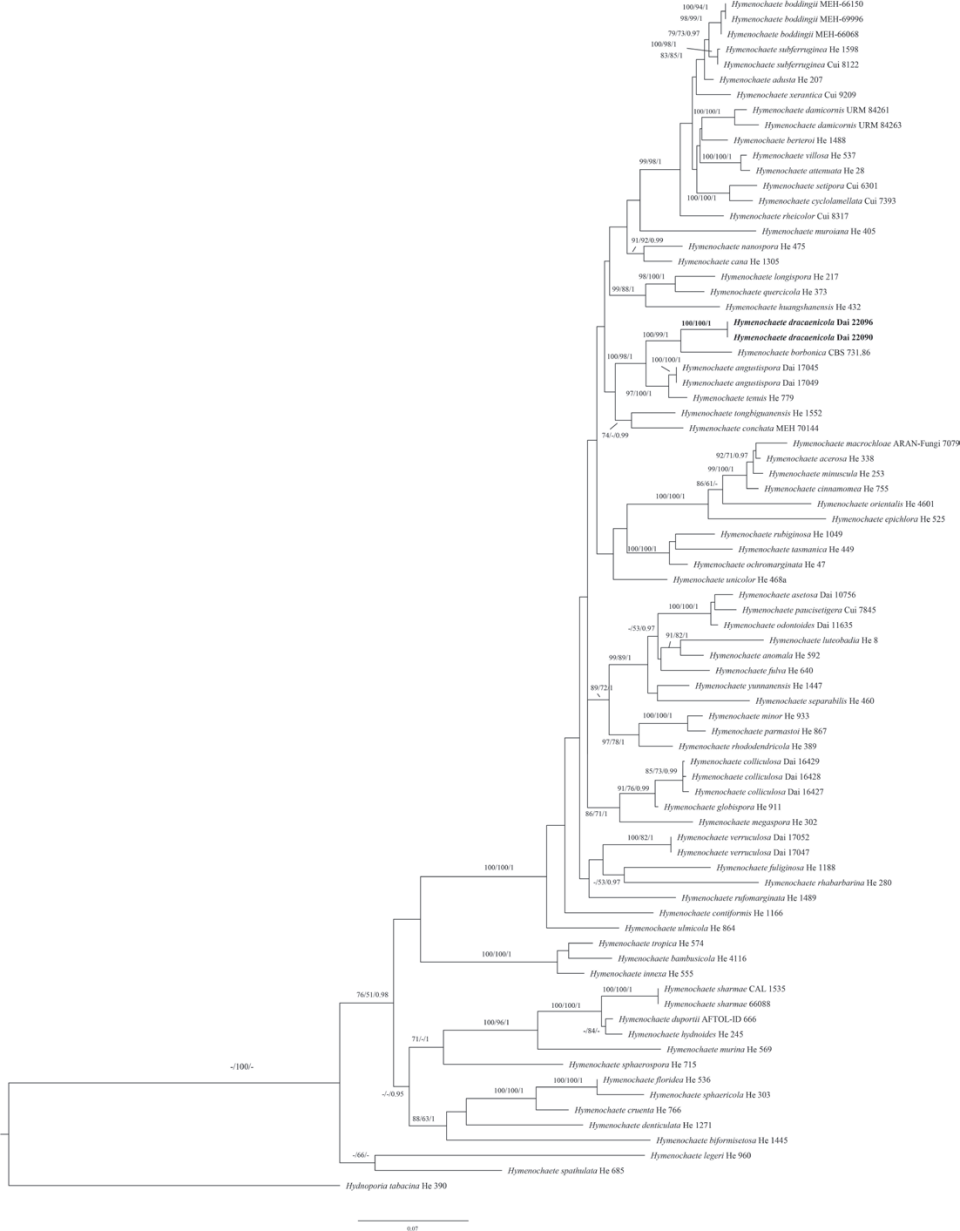
Phylogeny of *Hymenochaete* and related species by ML analysis based on combined ITS and nLSU rDNA sequences. Branches are labelled with maximum likelihood bootstrap > 70%, parsimony bootstrap proportions > 50%, and Bayesian posterior probabilities > 0.95, respectively. New species is in bold.

The phylogeny (Fig. 2) inferred from the ITS and nLSU sequences demonstrated that the new species *Hymenochaete dracaenicola* clustered in the *Hymenochaete* clade and two specimens of *H. dracaenicola* form a lineage with strong support (100% BS, 100% BP, 1.00 BPP, Fig. 2).

### Taxonomy

#### Fulvifomes dracaenicola

Taxon classificationFungiHymenochaetalesHymenochaetaceae

Z.B. Liu & Y.C. Dai sp. nov.

4648B76D-75FA-5D94-B7BB-6DC1AF828646

838682

Figs 3 , 4

##### Diagnosis.

*Fulvifomes dracaenicola* is characterised by perennial, pileate, triquetrous basidioma with yellowish brown fresh pores which becoming honey yellow with silk sheening upon drying, a dimitic hyphal system in trama and monomitic in context, subglobose basidiospores measuring 4.8–5 × 4–4.1 μm.

**Figure 3.**
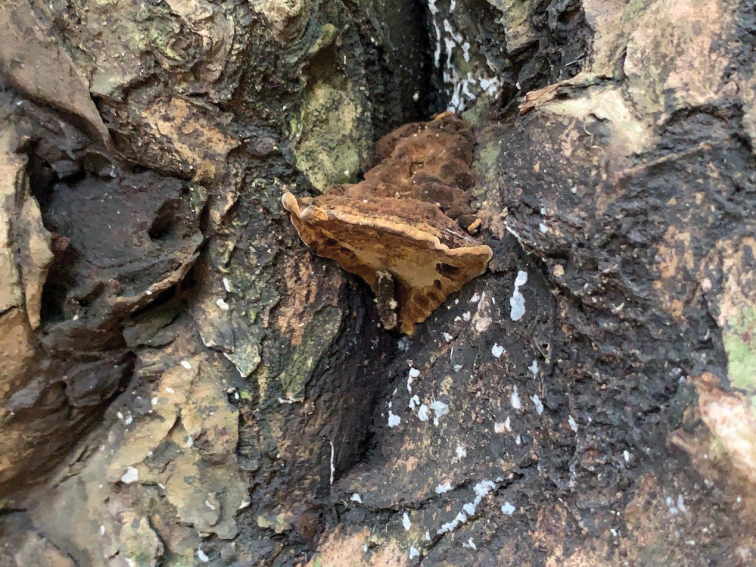
A basidiocarp of *Fulvifomes dracaenicola* (Holotype, Dai 22097). Scale bar: 1.0 cm. Photo by: Yu-Cheng Dai.

**Figure 4.**
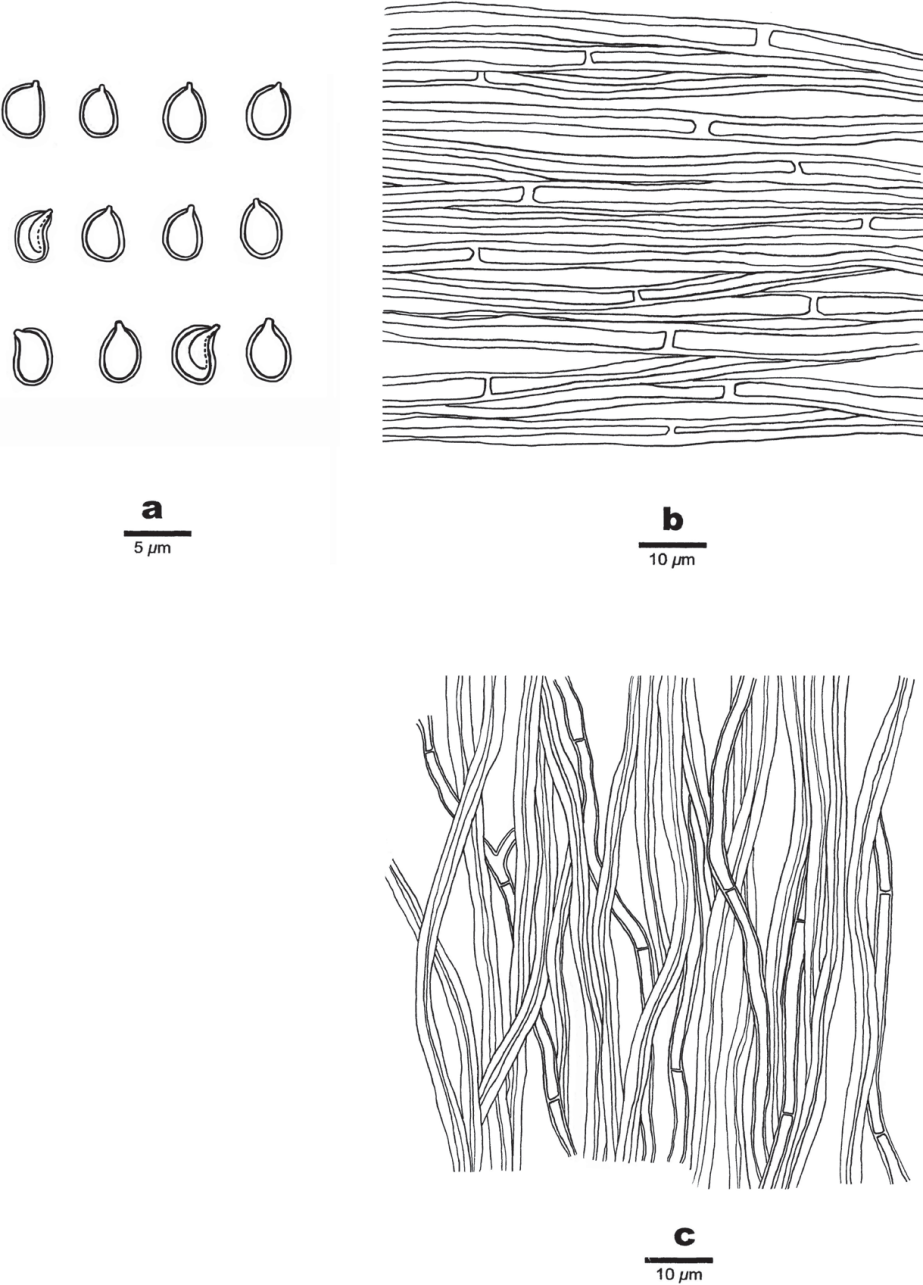
Microscopic structures of *Fulvifomes dracaenicola* (Holotype, Dai 22097) **a** basidiospores **b** hyphae of context **c** hyphae of the tubes. Drawings by: Meng Zhou.

##### Holotype.

China. Hainan Province, Sanya, Daxiaodongtian Park, N18.299, E109.172, on living tree of *Dracaena cambodiana*, 15.XI.2020, Dai 22097 (BJFC 035989).

##### Etymology.

*Dracaenicola* (Lat.): referring to the species growing on *Dracaena cambodiana*.

##### Fruiting body.

Basidioma perennial, pileate, without odor or taste and woody hard when fresh, light in weight when dry. Pilei triquetrous, projecting up to 2.5 cm, 2.3 cm wide and 2.6 cm thick at base. Pileal surface yellowish brown to grayish brown when fresh, vinaceous brown when dry, encrusted, glabrous, zonate, uncracked, margin olivaceous brown. Pore surface yellowish brown when fresh, honey yellow with silk sheening when dry; sterile margin indistinct; pores circular, 5–7 per mm; dissepiments thin, entire. Context cinnamon buff to fawn, corky, often darker near the pileus surface, up to 1.4 cm thick, with a distinct crust (black line) near pileus surface at the basal area, partly with additional crust (black line) within context or above tubes. Tubes cinnamon buff to cinnamon, woody hard, up to 1.2 cm thick, tube layers distinctly stratified, individual tube layer up to 0.5 cm long.

##### Hyphal structure.

Hyphal system dimitic in trama, monomitic in context; generative hyphae simple septate; tissues darkening but otherwise unchanged in KOH.

##### Context.

Generative hyphae apricot-orange to brownish-orange, thick-walled with a wide lumen, simple septate, unbranched, regularly arranged, 4.5–6 µm in diam.

##### Trama of the tubes.

Generative hyphae hyaline, thick-walled, simple septate, occasionally branched, 2–2.5 mm in diam; skeletal hyphae apricot-orange to brownish-orange, thick-walled to subsolid, unbranched, loosely interwoven, 3.5–4 mm in diam. Setae or setal hyphae absent; hymenium collapsed in the studied material, basidia and basidioles not seen.

##### Spores.

Basidiospores subglobose with an apiculus, yellowish brown, thick-walled, smooth, IKI–, CB–, occasionally collapsed when mature, 4.8–5(–5.5) × 4–4.1 μm, L = 5.02 μm, W = 4.04 μm, Q = 1.22–1.25 (n = 90/3).

##### Additional specimens (paratypes) examined.

China. Hainan Province, Sanya, Daxiaodongtian Park, N18.299, E109.172, on rotten wood of living *Dracaena cambodiana*, 15.XI.2020, Dai 22093 (BJFC 035986), Dai 22095 (BJFC 035987).

#### Hymenochaete dracaenicola

Taxon classificationFungiHymenochaetalesHymenochaetaceae

Z.B. Liu & Y.C. Dai sp. nov.

36A2207E-6F2E-5D13-9D2D-F13DDC27B60B

838683

Figs 5 , 6

##### Diagnosis.

*Hymenochaete dracaenicola* is characterised by annual, resupinate basidioma with a clay buff hymenophore, a dimitic hyphal system, absence of tomentum and cortex, subulate setae present in hyphal layer, absence of cystidia, presence of cystidioles and simple hyphidia, and oblong ellipsoid basidiospores measuring 5.2–5.8 × 2.5–2.8 µm.

**Figure 5.**
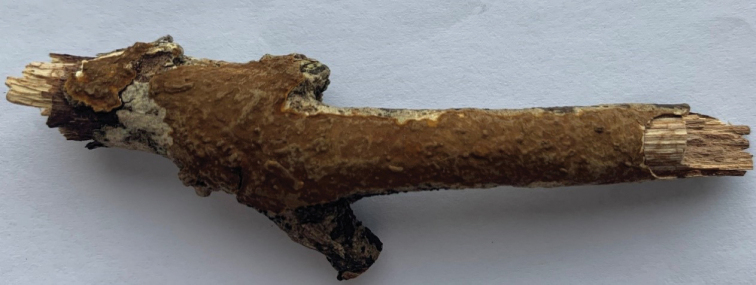
A basidiocarp of *Hymenochaete dracaenicola* (Holotype, Dai 22090). Scale bar: 1.0 cm. Photo by: Zhan-Bo Liu.

**Figure 6.**
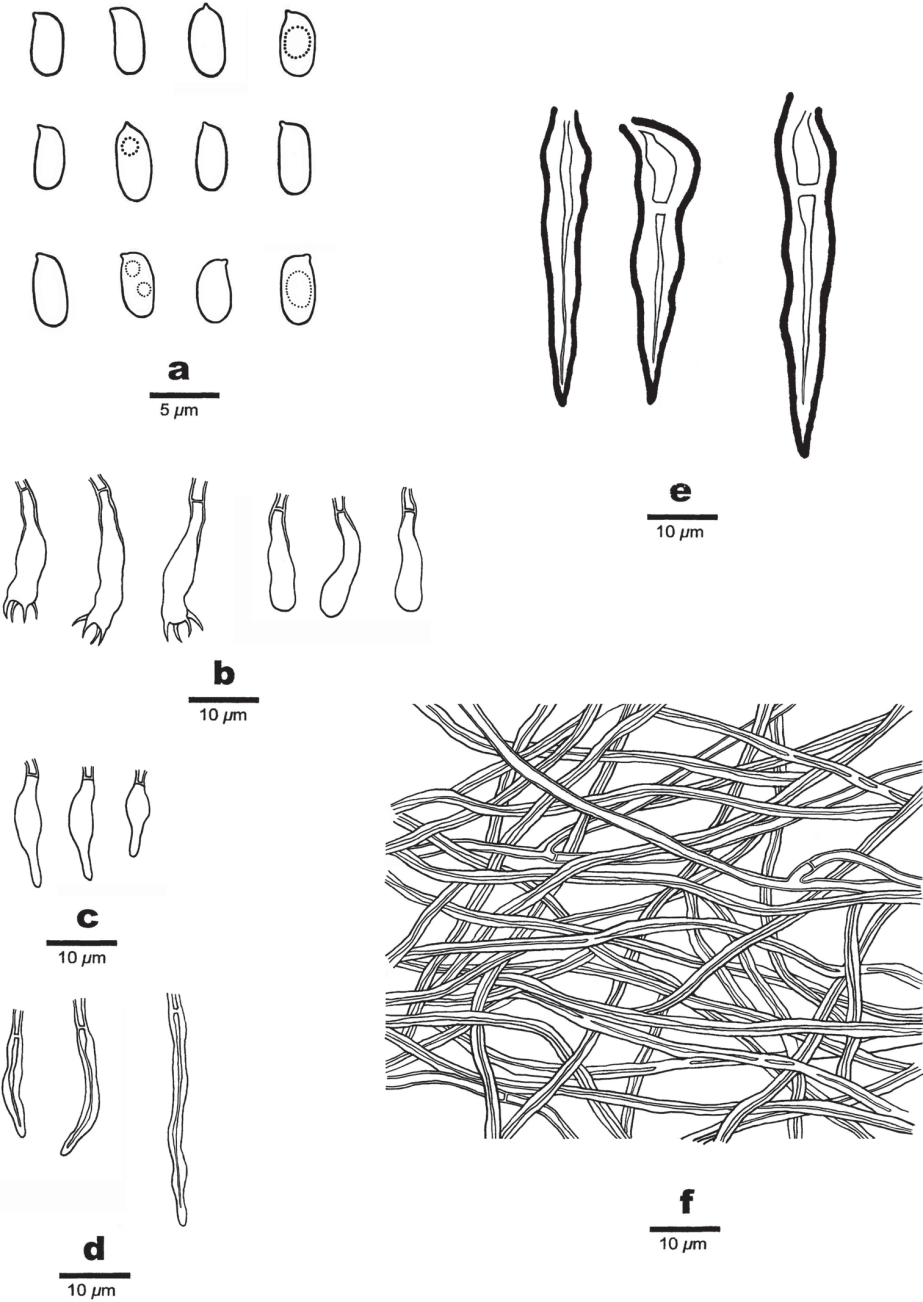
Microscopic structures of *Hymenochaete dracaenicola* (Holotype, Dai 22090) **a** basidiospores **b** basidia and basidioles **c** cystidioles **d** hyphidia **e** setae **f** Hyphae from hyphal layer. Drawings by: Meng Zhou.

##### Holotype.

China. Hainan Province, Sanya, Daxiaodongtian Park, N18.299, E109.172, on dead tree of *Dracaena cambodiana*, 15.XI.2020, Dai 22090 (BJFC 035983).

##### Etymology.

*Dracaenicola* (Lat.): referring to the species s growing on *Dracaena cambodiana*.

##### Fruiting body.

Basidioma annual, resupinate, adnate, not separable from substrate, hard corky, up to 7.5 cm long, 2 cm wide, and less than 0.1 mm thick at center. Hymenophore surface smooth or locally verruculose, clay buff, with some scattered crevices; margin cinnamon buff, up to 0.4 mm.

##### Hyphal structure.

Hyphal system dimitic; generative hyphae infrequent, simple septate; skeletal hyphae dominant; tissues darkening but otherwise unchanged in KOH.

##### Subiculum.

Tomentum and cortex absent; hyphal layer present. Generative hyphae infrequent, hyaline, thick-walled, simple septate, often branched, 1–2 µm in diam. Skeletal hyphae cinnamon to orange brown, thick-walled to subsolid, rarely branched, interwoven, 1.5–2.5 µm in diam.

##### Hymenium.

Hyphae similar to those in hyphal layer. Setal layer present, thickening with age, with one to several rows of overlapping setae. Setae numerous, subulate with blunt to acute tips, orange brown to reddish brown, smooth, occasionally with a hyphal sheath, distinctly thick-walled, 30–57 × 6–10 µm, embedded or projecting up to 35 µm beyond the hymenium. Cystidia absent; cystidioles present, fusoid, hyaline, thin-walled, basally swollen, with a sharp or often hyphoid neck, 10–17 × 2.5–4 μm; Simple hyphidia present, scattered, thick-walled, 15–36 × 2–3.5 µm. Basidia subclavate to subcylindrical, with walls thickening toward the base, with four sterigmata and a basal simple septum, 17–23(–25) × 3.5–5 µm; basidioles similar to basidia but smaller.

##### Spores.

Basidiospores oblong ellipsoid with an apiculus, hyaline, thin-walled, smooth, IKI–, CB–, occasionally bearing a guttule, (5–)5.2–5.8(–6.1) × 2.5–2.8 µm, L = 5.6 µm, W = 2.68 µm, Q = 2.03–2.15 (n = 60/2).

##### Additional specimen (paratype) examined.

China. China. Hainan Province, Sanya, Daxiaodongtian Park, N18.299, E109.172, on fallen branch of *Dracaena cambodiana*, 15.XI.2020, Dai 22096 (BJFC 035988).

## Discussion

*Fulvifomes dracaenicola* and *Hymenochaete dracaenicola* were found in tropical regions of China. It is interesting that both species growing on *Dracaena cambodiana*.

Morphologically, *Fulvifomes dracaenicola* (5–7 per mm) shares similar pores with *F. kawakamii* (M.J. Larsen, Lombard & Hodges) T. Wagner & M. Fisch. (5–7 per mm, Larsen et al. 1985), *F. robiniae* (5–6 per mm, Salvador-Montoya et al. 2018), *F. swieteniae* Murrill (5–7 per mm, Hattori et al. 2014) and *F. thailandicus* L.W. Zhou (6–7 per mm, Zhou 2015). *F. dracaenicola* and *F. kawakamii* share perennial, pileate basidioma, but basidioma of *F. dracaenicola* is solitary and glabrous, while basidioma of *F. kawakamii* is imbricate and nodulose. In addition, basidioma of *F. kawakamii* is much bigger (30–40 × 10–20 × 5–10 cm, Larsen et al. 1985) than that of *F. dracaenicola.* And basidiospores of *F. dracaenicola* are bigger than that of *F. kawakamii* (4.8–5 × 4–4.1 µm vs. 4.5 × 3.5 µm, Larsen et al. 1985). *F. dracaenicola* and *F. robiniae* share perennial, triquetrous and solitary basidioma, a monomitic hyphal system in context and dimitic in trama, however, *F. robiniae* can be distinguished from *F. dracaenicola* by its bigger basidiospores (5.5–6 × 4–5.5 µm vs. 4.8–5 × 4–4.1 µm). In addition, *F. dracaenicola* has a distinct crust (black line) on the pileal surface, but the crust (black line) is absent in *F. robiniae* (Gilbertson and Ryvarden 1987). *F. dracaenicola* resembles *F. swieteniae* by perennial, glabrous basidioma, a dimitic hyphal system in trama, however, basidioma of *F. swieteniae* is ungulate and pileal surface of *F. swieteniae* is azonate, while basidioma of *F. dracaenicola* is triquetrous and its pileal surface is zonate. And *F. dracaenicola* can be also distinguished from *F. swieteniae* by its wider basidiospores (4–4.1 µm vs. 3–4 µm, Salvador-Montoya et al. 2018). *F. dracaenicola* is similar to *F. thailandicus* by sharing perennial, solitary basidioma, but *F. thailandicus* can be distinguished from *F. dracaenicola* by a dimitic hyphal system in context, and its bigger basidiospores (5–5.8 × 4.1–4.8 µm vs. 4.8–5 × 4–4.1 µm, Zhou 2015).

Two specimens of *Fulvifomes dracaenicola* form a lineage with strong support (100% BP, 100% BS, 1.00 BPP, Fig. 1) in our phylogeny. *F. dracaenicola* is closely related to *F. siamensis* T. Hatt. et al. (98% BP, 99% BS, 1.00 BPP, Fig. 1) and both species share perennial, pileate basidioma, a dimitic hyphal system in trama and monomitic in context, and occurring in tropical Asia. Morphologically they can be easily differentiated by the presence of crust on the pileus surface. *F. dracaenicola* has a distinct crust (black line) on the pileus surface and partly with additional crust (black line) within context or above tubes, but the crust (black line) is absent in. *F. siamensis* (Hattori et al. 2014). Besides, *F. siamensis* has applanate pilei pores 7–8 per mm, thin- to thick-walled contextual generative hyphae 2–8 μm wide, and basidiospore 4–5 μm wide (Hattori et al. 2014), while *F. dracaenicola* has triquetrous pilei, pores 5–7 per mm, thick-walled contextual generative hyphae 4.5–6 μm wide, and basidiospore 4–4.1 μm wide. In addition, *F. siamensis* grows on *Xylocarpus granatum* in mangrove while *F. dracaenicola* grows on *Dracaena cambodiana* in terrestrial ecosystem.

Morphologically, to avoid redescribing the existed species, we review the monograph by Léger (1998) and compare *Hymenochaete dracaenicola* with all the species in the monograph. *H. dracaenicola* belongs to the section “FULTOCHAETE” and is similar to *H. epichlora* (Berk. & M.A. Curtis) Cooke. Both species share resupinate and adnate basidioma, absence of tomentum and cortex, similar setae (30–57 × 6–10 µm vs. 30–60 × 5.5–9 µm in *H. epichlora*, Cooke 1880), but *H. dracaenicola* has a dimitic hyphal system, while *H. epichlora* has a monomitic or subdimitic hyphal system and smaller basidiospores (5.2–5.8 × 2.5–2.8 µm vs. 3.5–5 × 1.8–2.5 µm, Cooke 1880). Besides, *H. dracaenicola* has simple hyphidia in hymenium, but hyphidia are absent in *H. epichlora* (Cooke 1880).Phylogenetically, two specimens of *Hymenochaete dracaenicola* form a lineage with strong support (100% BP, 100% BS, 1.00 BPP, Fig. 2). *H. dracaenicola* clusters together with *H. borbonica* J.C. Léger & Lanq., *H. angustispora* S.H. He & Y.C. Dai and *H. tenuis* Peck with strong support (100% BS, 98% BP, 1.00 BPP, Fig. 2). Morphologically, setae in *H. dracaenicola* are shorter than in *H. borbonica* (30–57 µm vs. 60–70 µm in *H. borbonica*). In addition, basidiospores of *H. dracaenicola* are oblong ellipsoid while they are suballantoid in *H. borbonica* (5–6 × 2 µm, Léger and Lanquetin 1987). *H. angustispora* is different from *H. dracaenicola* by a monomitic hyphal system and narrowly cylindrical to allantoid basidiospores (5–7 × 1.8–2.2 µm, He et al. 2017). *H. tenuis* can be distinguished from *H. dracaenicola* through its smaller basidiospores (4.5–5.5 × 2–2.5 µm vs. 5.2–5.8 × 2.5–2.8 µm) and a monomitic hyphal system (Peck 1887).

## Supplementary Material

XML Treatment for Fulvifomes dracaenicola

XML Treatment for Hymenochaete dracaenicola
